# Bis[3-(meth­oxy­carbon­yl)anilinium] hexa­chloridostannate(IV)

**DOI:** 10.1107/S1600536810054310

**Published:** 2011-01-15

**Authors:** Rui-Ting Xue, Xian-Wang Song, Shou-Gang Chen, Yan-Sheng Yin

**Affiliations:** a Institute of Material Science and Engineering, Ocean University of China, Qingdao, Shandong 266100, People’s Republic of China

## Abstract

In the title compound, (NH_3_C_6_H_4_CO_2_CH_3_)_2_[SnCl_6_], the anions are situated on inversion centers so the asymmetric unit contains one cation and one half-anion. In the crystal, inter­molecular N—H⋯Cl and N—H⋯O hydrogen bonds link the cations and anions into layers parallel to the *ac* plane. The crystal packing exhibits voids of 37 Å^3^.

## Related literature

For general background to inorganic–organic hybrid compounds, see: Cheetham *et al.* (1999 ▶); Descalzo *et al.* (2006 ▶); Sanchez *et al.* (2003 ▶, 2005 ▶).
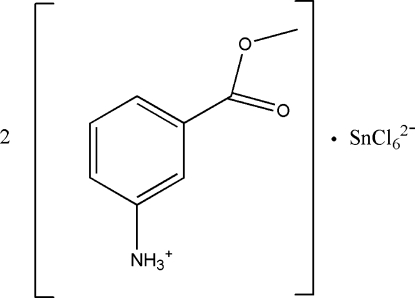


## Experimental

### 

#### Crystal data

(C_8_H_10_NO_2_)_2_[SnCl_6_]
*M* *_r_* = 635.73
Triclinic, 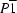

*a* = 7.2320 (7) Å
*b* = 8.2701 (9) Å
*c* = 11.2801 (12) Å
α = 86.980 (2)°
β = 81.970 (2)°
γ = 65.870 (1)°
*V* = 609.66 (11) Å^3^
*Z* = 1
Mo *K*α radiation
μ = 1.73 mm^−1^
*T* = 293 K
0.18 × 0.16 × 0.12 mm

#### Data collection

Bruker SMART CCD area-detector diffractometer
Absorption correction: multi-scan (*SADABS*; Sheldrick, 1996 ▶) *T*_min_ = 0.746, *T*_max_ = 0.819
3096 measured reflections
2084 independent reflections
1846 reflections with *I* > 2σ(*I*)
*R*_int_ = 0.032

#### Refinement

*R*[*F* ^2^ > 2σ(*F* ^2^)] = 0.057
*wR*(*F* ^2^) = 0.156
*S* = 1.01
2084 reflections
135 parameters
H-atom parameters constrained
Δρ_max_ = 3.34 e Å^−3^
Δρ_min_ = −1.32 e Å^−3^

### 

Data collection: *SMART* (Bruker, 1997 ▶); cell refinement: *SAINT* (Bruker, 1997 ▶); data reduction: *SAINT*; program(s) used to solve structure: *SHELXS97* (Sheldrick, 2008 ▶); program(s) used to refine structure: *SHELXL97* (Sheldrick, 2008 ▶); molecular graphics: *XP* in *SHELXTL* (Sheldrick, 2008 ▶); software used to prepare material for publication: *SHELXL97*.

## Supplementary Material

Crystal structure: contains datablocks global, I. DOI: 10.1107/S1600536810054310/cv5016sup1.cif

Structure factors: contains datablocks I. DOI: 10.1107/S1600536810054310/cv5016Isup2.hkl

Additional supplementary materials:  crystallographic information; 3D view; checkCIF report

## Figures and Tables

**Table 1:** Hydrogen-bond geometry (Å, °)

*D*—H⋯*A*	*D*—H	H⋯*A*	*D*⋯*A*	*D*—H⋯*A*
N1—H1*A*⋯O2^i^	0.89	1.99	2.832 (7)	157
N1—H1*B*⋯Cl1^i^	0.89	3.00	3.542 (6)	121
N1—H1*C*⋯Cl2^ii^	0.89	2.57	3.419 (6)	160
N1—H1*B*⋯Cl3^iii^	0.89	2.42	3.267 (6)	159

Symmetry codes: (i) 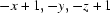; (ii) 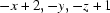; (iii) 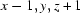.

**Fig. 1.**
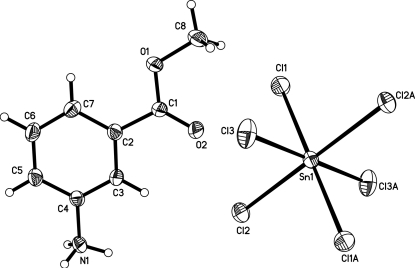
The structure of the title compound, showing 30% probability displacement ellipsoids and the atom-numbering scheme [symmetry code: (A) -x + 2, -y, -z)

## supplementary crystallographic information

### Comment

Inorganic-organic hybrid materials have been of great interest over recent years [Cheetham *et al.*, 1999]. The supramolecular chemistry, the optical properties and the applications of the inorganic-organic nanocomposites have been reviewed in the literatures [Descalzo *et al.*, 2006; Sanchez *et al.*, 2003, 2005]. Recently, we have prepared the title compound. Here we present its crystal structure.

The title compound contains SnCl_6_ inorganic anions and organic cations. The SnCl_6_ inorganic anion displays regular octahedron, with average Sn—Cl distance of 2.4073 Å.The angles of Cl—Sn—Cl are 89.45 to 90.95° for the chlorine atoms in *cis* positions. In the organic cation, the dihedral angle between the ester group and the phenyl ring is 5.7(0.3)°.

In the crystal structure, intermolecular N—H···Cl and N—H···O hydrogen bonds (Table 1) link cations and anions into layers parallel to *ac* plane.

### Experimental

3-aminobenzoic acid (10 mmol) was dissolved to methanol (10 ml) and 5 ml hydrochloric acid was added. A few minutes later, an methanol solution (10 ml) of tin tetrachloride (5 mmol) was added with stirring. The reaction mixture was stirred for 4 h, a yellow solid then separated out. The precipitate formed was filtered off, washed several times with anhydrous methanol and dried under vacuum. Yellow block crystals of the title compound were obtained from methanol solution after four days by slow evaporation at room temperature.

### Refinement

All H-atoms were positioned geometrically and refined using a riding model, with C—H = 0.96 Å (methyl), 0.93 Å (aromatic), N—H =0.89 Å (ammonium) and *U*_iso_(H) =1.5*U*_eq_(C), *U*_iso_(H) =1.2*U*_eq_(C),*U*_iso_(H) =1.52*U*_eq_(N). The highest residual peak of 3.34 e Å^-3^ is situated 1.75 Å at atom H1B.

### Crystal data

**Table:**

(C_8_H_10_NO_2_)_2_[SnCl_6_]	*Z* = 1
*M**_r_* = 635.73	*F*(000) = 314
Triclinic, *P*1	*D*_x_ = 1.732 Mg m^−^^3^
*a* = 7.2320 (7) Å	Mo *K*α radiation, λ = 0.71073 Å
*b* = 8.2701 (9) Å	Cell parameters from 2281 reflections
*c* = 11.2801 (12) Å	θ = 2.7–27.6°
α = 86.980 (2)°	µ = 1.73 mm^−^^1^
β = 81.970 (2)°	*T* = 293 K
γ = 65.870 (1)°	Block, colourless
*V* = 609.66 (11) Å^3^	0.18 × 0.16 × 0.12 mm

### Data collection

**Table:**

Bruker SMART CCD area-detector diffractometer	2084 independent reflections
Radiation source: fine-focus sealed tube	1846 reflections with *I* > 2σ(*I*)
graphite	*R*_int_ = 0.032
phi and ω scans	θ_max_ = 25.0°, θ_min_ = 1.8°
Absorption correction: multi-scan (*SADABS*; Sheldrick, 1996)	*h* = −8→8
*T*_min_ = 0.746, *T*_max_ = 0.819	*k* = −9→5
3096 measured reflections	*l* = −13→12

### Refinement

**Table:**

Refinement on *F*^2^	Primary atom site location: structure-invariant direct methods
Least-squares matrix: full	Secondary atom site location: difference Fourier map
*R*[*F*^2^ > 2σ(*F*^2^)] = 0.057	Hydrogen site location: inferred from neighbouring sites
*wR*(*F*^2^) = 0.156	H-atom parameters constrained
*S* = 1.01	*w* = 1/[σ^2^(*F*_o_^2^) + (0.121*P*)^2^] where *P* = (*F*_o_^2^ + 2*F*_c_^2^)/3
2084 reflections	(Δ/σ)_max_ < 0.001
135 parameters	Δρ_max_ = 3.34 e Å^−^^3^
0 restraints	Δρ_min_ = −1.32 e Å^−^^3^

### Special details

**Table:**

Geometry. All e.s.d.'s (except the e.s.d. in the dihedral angle between two l.s. planes) are estimated using the full covariance matrix. The cell e.s.d.'s are taken into account individually in the estimation of e.s.d.'s in distances, angles and torsion angles; correlations between e.s.d.'s in cell parameters are only used when they are defined by crystal symmetry. An approximate (isotropic) treatment of cell e.s.d.'s is used for estimating e.s.d.'s involving l.s. planes.
Refinement. Refinement of *F*^2^ against ALL reflections. The weighted *R*-factor *wR* and goodness of fit *S* are based on *F*^2^, conventional *R*-factors *R* are based on *F*, with *F* set to zero for negative *F*^2^. The threshold expression of *F*^2^ > σ(*F*^2^) is used only for calculating *R*-factors(gt) *etc*. and is not relevant to the choice of reflections for refinement. *R*-factors based on *F*^2^ are statistically about twice as large as those based on *F*, and *R*- factors based on ALL data will be even larger.

### Fractional atomic coordinates and isotropic or equivalent isotropic displacement parameters (Å^2^)

**Table:**

	*x*	*y*	*z*	*U*_iso_*/*U*_eq_	
Sn1	1.0000	0.0000	0.0000	0.0328 (3)	
Cl1	0.6515 (2)	0.2167 (2)	0.03141 (14)	0.0513 (5)	
Cl2	0.9962 (3)	−0.0365 (2)	0.21342 (12)	0.0508 (4)	
Cl3	1.1248 (3)	0.2268 (2)	0.00885 (15)	0.0552 (5)	
N1	0.5099 (9)	0.0834 (7)	0.7957 (4)	0.0449 (13)	
H1A	0.5244	−0.0115	0.7554	0.067*	
H1B	0.4246	0.0948	0.8628	0.067*	
H1C	0.6309	0.0709	0.8138	0.067*	
O1	0.2054 (7)	0.4933 (6)	0.3593 (4)	0.0454 (10)	
O2	0.3547 (9)	0.2009 (6)	0.3694 (4)	0.0652 (15)	
C1	0.2955 (9)	0.3441 (8)	0.4139 (5)	0.0390 (13)	
C2	0.3176 (9)	0.3708 (8)	0.5388 (5)	0.0374 (13)	
C3	0.3979 (9)	0.2212 (8)	0.6087 (5)	0.0392 (13)	
H3	0.4309	0.1090	0.5783	0.047*	
C4	0.4274 (9)	0.2416 (8)	0.7219 (5)	0.0357 (12)	
C5	0.3753 (10)	0.4048 (8)	0.7713 (5)	0.0426 (14)	
H5	0.3942	0.4151	0.8500	0.051*	
C6	0.2944 (11)	0.5536 (9)	0.7020 (6)	0.0473 (15)	
H6	0.2596	0.6652	0.7339	0.057*	
C7	0.2650 (9)	0.5377 (8)	0.5864 (6)	0.0424 (14)	
H7	0.2100	0.6382	0.5400	0.051*	
C8	0.1833 (12)	0.4799 (10)	0.2347 (6)	0.0552 (18)	
H8A	0.3156	0.4360	0.1879	0.083*	
H8B	0.1003	0.5948	0.2062	0.083*	
H8C	0.1193	0.4000	0.2277	0.083*	

### Atomic displacement parameters (Å^2^)

**Table:**

	*U*^11^	*U*^22^	*U*^33^	*U*^12^	*U*^13^	*U*^23^
Sn1	0.0336 (4)	0.0345 (4)	0.0272 (3)	−0.0093 (3)	−0.0072 (2)	−0.0035 (2)
Cl1	0.0371 (8)	0.0534 (10)	0.0467 (9)	0.0012 (7)	−0.0095 (6)	−0.0131 (7)
Cl2	0.0680 (11)	0.0481 (9)	0.0257 (8)	−0.0119 (8)	−0.0085 (6)	−0.0018 (6)
Cl3	0.0693 (11)	0.0552 (10)	0.0520 (9)	−0.0379 (9)	0.0033 (8)	−0.0157 (8)
N1	0.062 (3)	0.044 (3)	0.027 (2)	−0.019 (3)	−0.012 (2)	0.000 (2)
O1	0.056 (3)	0.040 (2)	0.032 (2)	−0.009 (2)	−0.0126 (18)	0.0018 (18)
O2	0.108 (4)	0.037 (3)	0.037 (2)	−0.011 (3)	−0.023 (3)	−0.003 (2)
C1	0.046 (3)	0.033 (3)	0.033 (3)	−0.010 (3)	−0.008 (2)	0.000 (3)
C2	0.037 (3)	0.040 (3)	0.032 (3)	−0.012 (3)	−0.003 (2)	−0.005 (2)
C3	0.044 (3)	0.036 (3)	0.032 (3)	−0.010 (3)	−0.003 (2)	−0.008 (2)
C4	0.038 (3)	0.039 (3)	0.027 (3)	−0.014 (3)	−0.002 (2)	−0.001 (2)
C5	0.047 (3)	0.046 (4)	0.033 (3)	−0.017 (3)	−0.004 (2)	−0.008 (3)
C6	0.060 (4)	0.039 (3)	0.040 (3)	−0.019 (3)	−0.001 (3)	−0.010 (3)
C7	0.045 (3)	0.034 (3)	0.043 (3)	−0.012 (3)	0.001 (3)	−0.003 (3)
C8	0.063 (4)	0.055 (4)	0.035 (3)	−0.010 (3)	−0.013 (3)	0.005 (3)

### Geometric parameters (Å, °)

**Table:**

Sn1—Cl3	2.4028 (16)	C2—C3	1.388 (9)
Sn1—Cl3^i^	2.4028 (16)	C2—C7	1.389 (9)
Sn1—Cl2^i^	2.4081 (14)	C3—C4	1.354 (8)
Sn1—Cl2	2.4081 (14)	C3—H3	0.9300
Sn1—Cl1^i^	2.4111 (15)	C4—C5	1.371 (9)
Sn1—Cl1	2.4111 (15)	C5—C6	1.381 (9)
N1—C4	1.465 (7)	C5—H5	0.9300
N1—H1A	0.8900	C6—C7	1.372 (9)
N1—H1B	0.8900	C6—H6	0.9300
N1—H1C	0.8900	C7—H7	0.9300
O1—C1	1.305 (7)	C8—H8A	0.9600
O1—C8	1.451 (7)	C8—H8B	0.9600
O2—C1	1.195 (7)	C8—H8C	0.9600
C1—C2	1.477 (8)		
			
Cl3—Sn1—Cl3^i^	180.00 (3)	C3—C2—C7	119.9 (5)
Cl3—Sn1—Cl2^i^	90.55 (6)	C3—C2—C1	117.6 (5)
Cl3^i^—Sn1—Cl2^i^	89.45 (6)	C7—C2—C1	122.4 (6)
Cl3—Sn1—Cl2	89.45 (6)	C4—C3—C2	118.8 (5)
Cl3^i^—Sn1—Cl2	90.55 (6)	C4—C3—H3	120.6
Cl2^i^—Sn1—Cl2	180.00 (9)	C2—C3—H3	120.6
Cl3—Sn1—Cl1^i^	89.05 (7)	C3—C4—C5	122.5 (6)
Cl3^i^—Sn1—Cl1^i^	90.95 (7)	C3—C4—N1	118.5 (5)
Cl2^i^—Sn1—Cl1^i^	89.55 (6)	C5—C4—N1	119.0 (5)
Cl2—Sn1—Cl1^i^	90.45 (6)	C4—C5—C6	118.7 (5)
Cl3—Sn1—Cl1	90.95 (7)	C4—C5—H5	120.7
Cl3^i^—Sn1—Cl1	89.05 (7)	C6—C5—H5	120.7
Cl2^i^—Sn1—Cl1	90.45 (6)	C7—C6—C5	120.4 (6)
Cl2—Sn1—Cl1	89.55 (6)	C7—C6—H6	119.8
Cl1^i^—Sn1—Cl1	180.00 (6)	C5—C6—H6	119.8
C4—N1—H1A	109.5	C6—C7—C2	119.7 (6)
C4—N1—H1B	109.5	C6—C7—H7	120.1
H1A—N1—H1B	109.5	C2—C7—H7	120.1
C4—N1—H1C	109.5	O1—C8—H8A	109.5
H1A—N1—H1C	109.5	O1—C8—H8B	109.5
H1B—N1—H1C	109.5	H8A—C8—H8B	109.5
C1—O1—C8	116.2 (5)	O1—C8—H8C	109.5
O2—C1—O1	124.6 (5)	H8A—C8—H8C	109.5
O2—C1—C2	123.0 (5)	H8B—C8—H8C	109.5
O1—C1—C2	112.5 (5)		

Symmetry codes: (i) −*x*+2, −*y*, −*z*.

### Hydrogen-bond geometry (Å, °)

**Table:**

*D*—H···*A*	*D*—H	H···*A*	*D*···*A*	*D*—H···*A*
N1—H1A···O2^ii^	0.89	1.99	2.832 (7)	157.
N1—H1B···Cl1^ii^	0.89	3.00	3.542 (6)	121.
N1—H1C···Cl2^iii^	0.89	2.57	3.419 (6)	160.
N1—H1B···Cl3^iv^	0.89	2.42	3.267 (6)	159.

Symmetry codes: (ii) −*x*+1, −*y*, −*z*+1; (iii) −*x*+2, −*y*, −*z*+1; (iv) *x*−1, *y*, *z*+1.
